# Supplementary material for: Cerebrospinal fluid-contacting neurons are sensory neurons with uniform morphological and region-specific electrophysiological properties in the mouse spinal cord
Source: Commun Biol. 2025 Aug 15;8:1233. doi: 10.1038/s42003-025-08559-x (PMC12356878; doi:10.1038/s42003-025-08559-x)
Supplement: Supplementary file 2 — Supplementary Information [file 42003_2025_8559_MOESM2_ESM.pdf]

Cerebrospinal Fluid-contacting neurons are sensory neurons with uniform morphological and region-specific electrophysiological properties in the mouse spinal cord

CROZAT Elysa*, BLASCO Edith*, RAMIREZ-FRANCO Jorge*, RIONDEL Priscille, JURČIĆ Nina, SEDDIK Riad, MICHELLE Caroline, TROUSLARD Jérôme & WANAVERBECQ Nicolas#

Institut de Neurosciences de la Timone, Aix Marseille Université (AMU) & CNRS, UMR7289, Marseille, France.

* CE, BE & RFJ Contributed equally to this work as first authors.
# Correspondence should be addressed to: nicolas.wanaverbecq@univ-amu.fr (WN)

Supplementary Figures

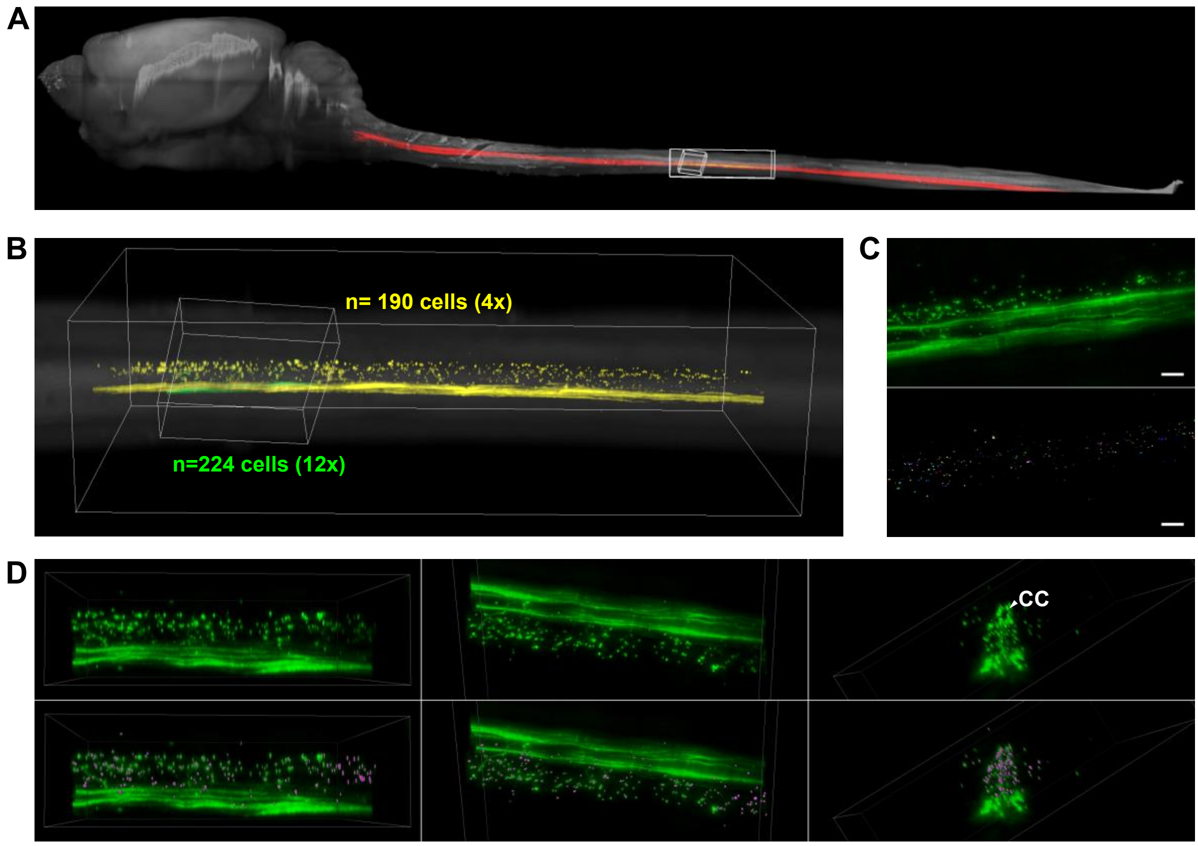

Supplementary Figure 1 - Comparison of the cell segmentation workflow and estimation of cell number at different light-sheet resolutions.

A) Full view corresponding to ***Fig. 1A***, showing regions acquired at different light sheet resolutions (1x, full view; 4x, large box; 12x small box). **B)** Detailed view of the thoracic region boxed in ***Supplementary Fig. 1A***, showing the estimation for cell numbers at 4x (yellow) and 12x (green) in equivalent light-sheet microscopy stacks. Boxes sizes are in µm along the Rostro-caudal (RC) x Latero-Median (LM) x Dorso-Ventral (DV): 4x image (yellow) 3076.13 x 1784.25 x 818; 12x image (green) 692.90 x 703.95 x 211.25. **C)** Maximal projection of the region boxed in ***Supplementary Fig. 1B*** in fluorescence (***Top***) and particle analysis of this same region (***Bottom***). Scale bar=50 µm. **D)** 3D views of the same region of the maximal projection depicted in **C** from lateral (***Left***), top (***Middle***), and front views (***Right***). Fluorescence images are depicted in green (***Top row***) and the result of the particle analysis workflow is merged in magenta (***Bottom row***). cc: Central canal. Scale: see Panel 1B.

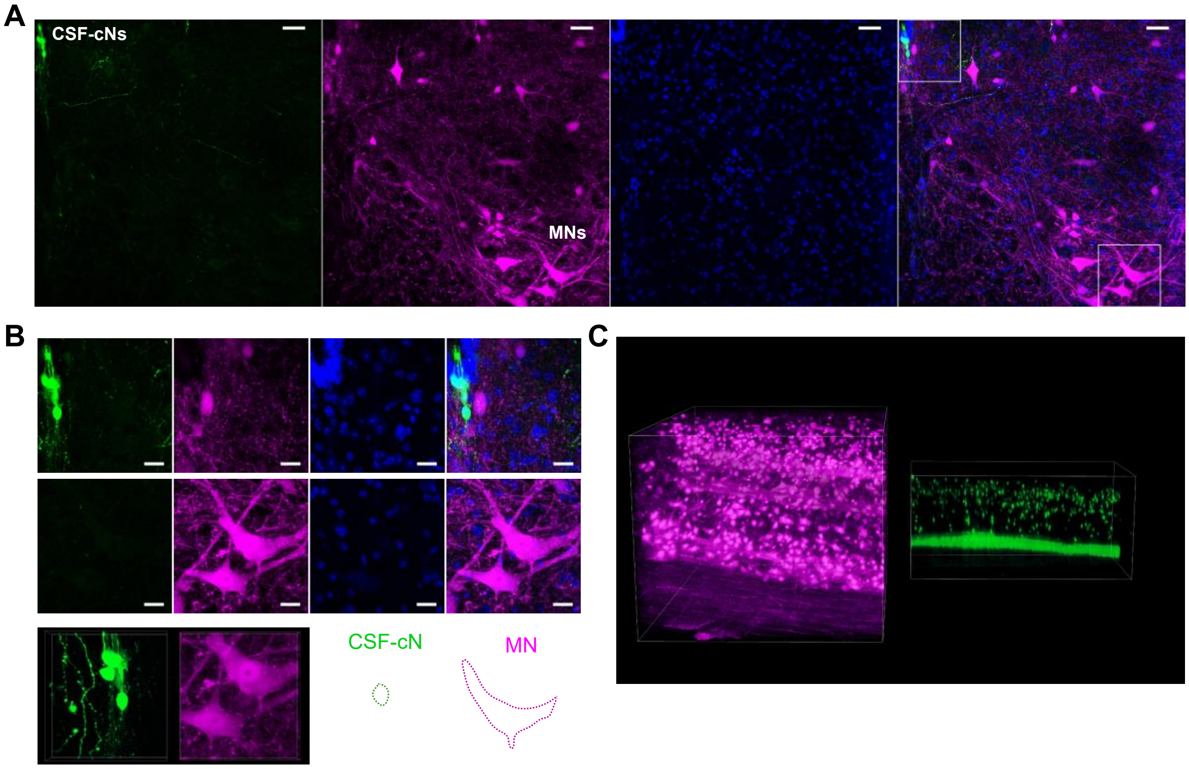

Supplementary Figure 2 - Size comparison of CSF-cNs to motor neurons using different microscopy techniques.

A) Confocal images of a large field of view showing CSF-cNs expressing EGFP (green) in a ChAT-Cre::tdTomato mouse ($ChAT^{+}$, magenta and DAPI, blue; Merge image, ***Right***). Expression of EGFP is triggered by intra cerebroventricular (icv) injections of AAV1-hSyn-EGFP particles as previously described. Scale bar=50 µm. **B)** Detailed views of the field of view shown in Panel A corresponding to the boxes depicted in the merge image of Panel of A (***Top Right***). Scale bar=20 µm. ***Bottom, Left.*** 3D renderings of the neurons depicted in ***Top*** panels. ***Bottom, Right***. silhouette of one CSF-cN (green) and one motor neuron (MN, magenta) for size comparison. **C)** Light-sheet microscopy images obtained at the highest possible magnification of microscope in one ChAT-Cre::tdTomato animal showing MNs and other $ChAT^{+}$ neurons (magenta), and in one Pkd2l1-Cre::tdTomato animal showing CSF-cNs (green). Note that even at the highest magnification, the resolution of the light-sheet microscope combined to the DISCO clearing technique remains poor to resolve individual CSF-cN somatas. Boxes sizes are (RC x LM x DV in µm); for $ChAT^{+}$ neurons: 555 x 752 x 468; for CSF-cNs: 565 x 285 x 266.

Supplementary Figure 3 - Transcriptomic profile of ionic conductances, voltage-gated channels and ligand-gated receptors of CSF-cNs in the mouse spinal cord.

Adapted from Yue et al., 2024 (Reference [11]). **Data were Accession:** GSE255883 ID: 200255883
https://www.ncbi.nlm.nih.gov/geo/query/acc.cgi?acc=GSE255883

For the detail about experimental procedure, refer to b in the original the publication:

Endogenous opioid signalling regulates spinal ependymal cell proliferation
Wendy W. S. Yue, Kouki K. Touhara, Kenichi Toma, Xin Duan & David Julius
Nature | Vol 634 | 10 October 2024

Raster plots with heat maps for gene expression in spinal CSF-cNs For each gene, the corresponding protein name of the subunit is mentioned. (**A**) Polycystine Transient Receptor Potentials (TRPPs) and Acid Sensing Ionic Conductances (ASICs). Sodium- (Nav; **B**), Potassium- (Kvs, Kirs and K(Ca); **C**) and Calcium- (Cav; **D**) voltage-gated channels. (**E**) Ionotropic and metabotropic GABAergic receptors. (**F**) Ionotropic glycinergic receptors. (**G**) Ionotropic and metabotropic glutamatergic receptors. (**H**) Ionotropic and metabotropic cholinergic receptors. (**I**) Ionotropic and metabotropic purinergic receptors.

A) Polycystine TRPs (TRPPs) and ASIC

	Proteins	Genes	Sample 1	Sample 2	Sample 3	Sample 4
TRPPs	PKD2/TRPP2	Pkd2	10.82	9.78	10.38	7.50
	PKD2L1/TRPP3	Pkd2l1	10.68	4.19	15.90	15.94
	PKD2L2/TRPP5	Pkd2l2	8.49	4.26	8.03	6.92
ASICs	ASIC1a/1b	Asic1	0.00	5.89	9.02	9.12
	ASIC2a	Asic2	0.00	0.00	8.86	0.00
	ASIC3	Asic3	0.00	0.00	0.00	0.00
	ASIC4	Asic4	0.00	0.00	8.99	7.98
	ASIC5	Asic5	0.00	0.00	0.00	0.00

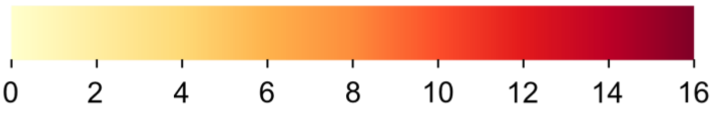

B) Sodium voltage-dependent channels

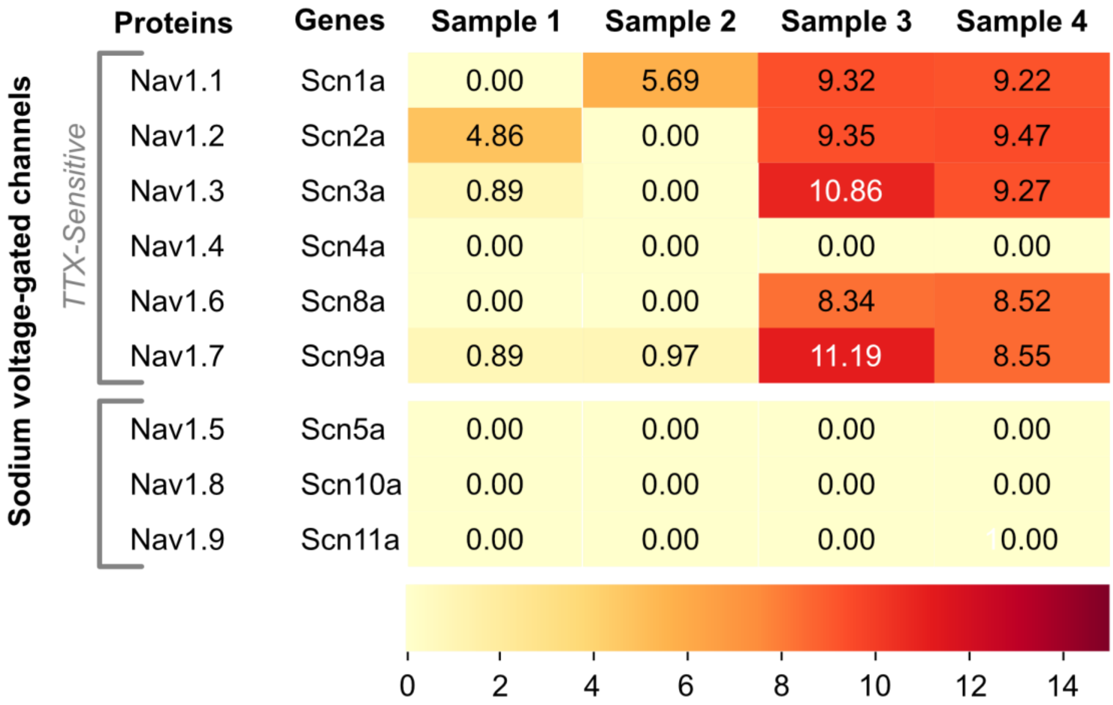

C) Potassium voltage-dependent channels

Potassium voltage-gated Channels (Kvs, 1)

Proteins	Genes	Sample 1	Sample 2	Sample 3	Sample 4
Kv1.1	Kcna1	5.47	8.21	0.00	8.41
Kv1.2	Kcna2	8.05	0.00	7.40	8.14
Kv1.3	Kcna3	8.50	0.97	8.81	9.62
Kv1.4	Kcna4	4.53	0.97	6.99	7.46
Kv1.5	Kcna5	0.00	0.00	7.54	8.70
Kv1.6	Kcna6	0.00	0.00	6.34	4.61
Kv1.7	Kcna7	0.00	4.19	0.00	0.00
Kv1.8	Kcna10	0.00	0.00	0.00	0.00
Kv2.1	Kcnb1	2.39	3.85	9.56	9.84
Kv2.2	Kcnb2	4.30	1.54	8.55	0.00
Kv3.1	Kcnc1	0.00	0.97	6.59	8.71
Kv3.2	Kcnc2	7.71	5.77	6.26	7.56
Kv3.3	Kcnc3	0.00	2.94	2.66	6.47
Kv3.4	Kcnc4	0.00	0.00	7.99	0.00
Kv4.1	Kcnd1	11.30	10.75	10.87	12.29
Kv4.2	Kcnd2	2.14	0.00	0.00	1.01
Kv4.3	Kcnd3	0.00	0.00	5.93	0.00
Kv5.1	Kcnf1	0.00	0.97	0.00	0.00
Kv6.1	Kcng1	0.00	0.00	0.00	0.00
Kv6.2	Kcng2	0.89	7.18	5.98	0.00
Kv6.3	Kcng3	2.96	0.97	.0.00	3.61
Kv6.4	Kcng4	9.27	0.97	0.00	0.00

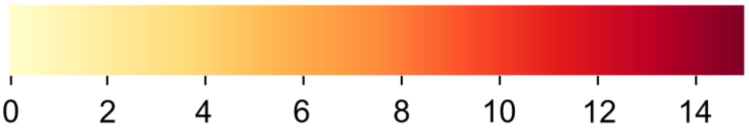

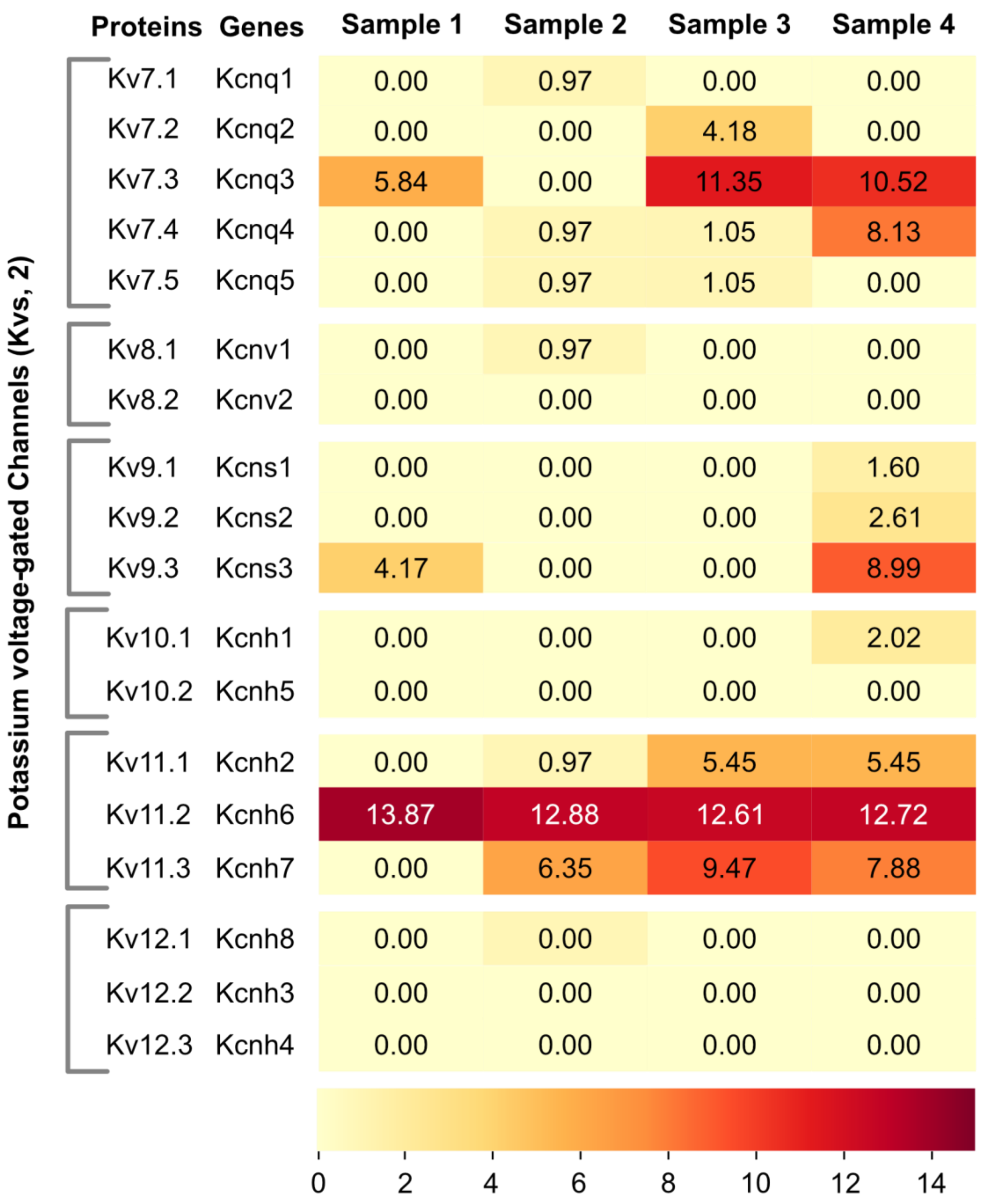
Potassium voltage-gated Channels (Kvs, 2)
Proteins Genes Sample 1 Sample 2 Sample 3 Sample 4
Kv7.1 Kcnq1 0.00 0.97 0.00 0.00
Kv7.2 Kcnq2 0.00 0.00 4.18 0.00
Kv7.3 Kcnq3 5.84 0.00 11.35 10.52
Kv7.4 Kcnq4 0.00 0.97 1.05 8.13
Kv7.5 Kcnq5 0.00 0.97 1.05 0.00
Kv8.1 Kcnv1 0.00 0.97 0.00 0.00
Kv8.2 Kcnv2 0.00 0.00 0.00 0.00
Kv9.1 Kcns1 0.00 0.00 0.00 1.60
Kv9.2 Kcns2 0.00 0.00 0.00 2.61
Kv9.3 Kcns3 4.17 0.00 0.00 8.99
Kv10.1 Kcnh1 0.00 0.00 0.00 2.02
Kv10.2 Kcnh5 0.00 0.00 0.00 0.00
Kv11.1 Kcnh2 0.00 0.97 5.45 5.45
Kv11.2 Kcnh6 13.87 12.88 12.61 12.72
Kv11.3 Kcnh7 0.00 6.35 9.47 7.88
Kv12.1 Kcnh8 0.00 0.00 0.00 0.00
Kv12.2 Kcnh3 0.00 0.00 0.00 0.00
Kv12.3 Kcnh4 0.00 0.00 0.00 0.00
0
2
4
6
8
10
12
14

		Proteins	Genes	Sample 1	Sample 2	Sample 3	Sample 4
Voltage-Gated inward rectifying K Channels (Kirs)		Kir1.1	Kcnj1	0.00	0.00	0.00	0.00
		Kir2.1	Kcnj2	10.06	12.22	10.51	10.75
		Kir3.1	Kcnj3	0.00	0.00	5.74	0.00
		Kir2.3	Kcnj4	0.00	0.00	0.00	0.00
		Kir3.4	Kcnj5	0.00	0.00	6.69	9.97
		Kir3.2	Kcnj6/7	0.00	0.00	7.54	5.45
		Kir6.1	Kcnj8	0.00	0.00	0.00	0.00
		Kir13.3	Kcnj9	0.00	8.68	8.67	8.54
		Kir1.2/4.1	Kcnj10	11.37	9.84	11.83	6.15
		Kir6.2	Kcnj11	0.00	0.00	0.00	0.00
		Kir2.2	Kcnj12	0.00	8.60	1.65	8.41
		Kir7.1	Kcnj13	7.78	0.00	7.93	1.01
		Kir2.4	Kcnj14	0.00	0.00	0.00	0.00
		Kir4.2	Kcnj15	0.00	0.00	0.00	0.00
		Kir5.1	Kcnj16	0.00	0.00	7.52	0.00
Voltage-Gated Calcium-activated K Channels	*Ca-activated*	KCa1.1	Kcnma1	12.10	11.85	11.11	11.45
		KCa2.1	Kcnn1	5.39	0.00	0.00	3.48
		KCa2.2	Kcnn2	0.00	0.00	0.00	0.00
		KCa2.3	Kcnn3	0.00	0.00	3.41	7.54
		KCa2.4	Kcnn4	8.04	7.04	7.85	0.00
		KCa5.1	Kcnu1	8.85	0.97	9.11	9.57
	Na-activated	KCa4.1	Kcnt1	0.00	0.00	2.07	0.00
		KCa4.2	Kcnt2	0.00	0.00	8.23	10.00

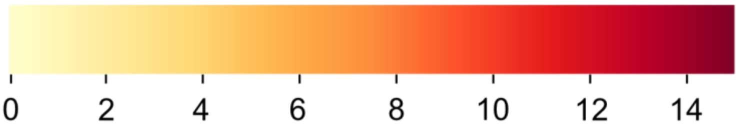

D) Calcium voltage-dependent channels and accessory subunits

Group	Type	Proteins	Genes	Sample 1	Sample 2	Sample 3	Sample 4
Voltage-Gated Ca Channels alpha Subunits	L Type	Cav1.1	Cacna1s	7.45	8.28	6.96	9.70
	L Type	Cav1.2	Cacna1c	4.68	9.18	7.13	6.35
	L Type	Cav1.3	Cacna1d	9.12	11.45	11.07	11.39
	L Type	Cav1.4	Cacna1f	0.00	0.00	6.12	0.00
	P/Q Type	Cav2.1	Cacna1a	3.49	5.07	8.13	6.62
	N Type	Cav2.2	Cacna1b	0.00	0.00	8.00	2.83
	R Type	Cav2.3	Cacna1e	1.43	0.00	10.91	8.90
	T Type	Cav3.1	Cacna1g	2.80	0.00	5.49	7.61
	T Type	Cav3.2	Cacna1h	0.00	0.00	2.07	1.01
	T Type	Cav3.3	Cacna1i	0.00	0.00	6.43	8.39
Calcium channels accessory subunits	α2δ Subunits	α2δ1	Cacna2d1	0.89	1.54	0.00	1.01
	α2δ Subunits	α2δ2	Cacna2d2	0.00	0.00	4.00	5.52
	α2δ Subunits	α2δ3	Cacna2d3	3.25	1.54	11.45	10.03
	α2δ Subunits	α2δ4	Cacna2d4	0.00	0.00	0.00	0.00
	β Subunits	β1	Cacnb1	7.31	7.35	8.68	0.00
	β Subunits	β2	Cacnb2	8.93	9.76	10.06	10.62
	β Subunits	β3	Cacnb3	7.24	0.00	10.25	1.01
	β Subunits	β4	Cacnb4	12.13	3.40	12.22	10.17
	γ Subunits	γ1	Cacng1	0.00	0.00	0.00	0.00
	γ Subunits	γ2	Cacng2	0.00	0.00	6.66	1.01
	γ Subunits	γ3	Cacng3	0.00	8.06	0.00	0.00
	γ Subunits	γ4	Cacng4	0.00	0.00	7.60	7.52
	γ Subunits	γ5	Cacng5	7.02	0.00	0.00	7.67
	γ Subunits	γ6	Cacng6	0.00	0.00	0.00	0.00
	γ Subunits	γ6.1	Cacng6_1	0.00	0.00	0.00	0.00
	γ Subunits	γ7	Cacng7	0.00	0.00	5.09	1.01
	γ Subunits	γ7.1	Cacng7_1	0.00	0.00	0.00	1.01
	γ Subunits	γ8	Cacng8	0.00	0.00	0.00	3.34
	γ Subunits	γ8.1	Cacng8_1	0.00	0.00	0.00	3.34

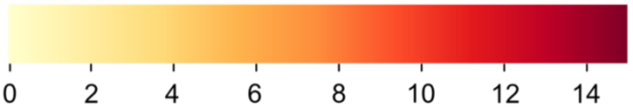

E) Ionotropic and metabotropic GABA receptors

		Proteins	Genes	Sample 1	Sample 2	Sample 3	Sample 4
$GABA_A$ Subunits	α Subunits	α1	Gabra1	0.00	0.97	9.52	11.98
		α2	Gabra2	0.00	6.20	10.26	11.00
		α3	Gabra3	0.00	0.97	8.13	8.89
		α4	Gabra4	0.00	0.00	1.05	10.21
	β Subunits	β2	Gabrb2	0.00	0.00	5.68	0.00
		β3	Gabrb3	7.95	0.00	7.61	9.59
		ε	Gabre	0.00	0.00	1.05	0.00
	γ Subunits	γ1	Gabrg1	0.00	0.00	9.42	0.00
		γ2	Gabrg2	0.00	0.00	10.45	10.55
		γ3	Gabrg3	0.00	0.00	7.08	8.72
		π	Gabrp	0.00	0.00	0.00	0.00
	ρ Subunits	ρ1	Gabrr1	0.00	0.00	0.00	0.00
		ρ2	Gabrr2	0.00	0.00	5.90	0.00
		ρ3	Gabrr3	0.00	0.00	0.00	0.00
$GABA_B$ Subunits	Gb Subunits	Gb1a	Gabbr1	9.20	7.79	10.24	10.19
		Gb1b	Gabbr1_1	4.82	6.04	5.05	6.52
		Gb1c	Gabbr1_2	6.40	3.94	6.05	7.93
		Gb1d	Gabbr1_3	6.40	3.94	6.05	7.93
		Gb2	Gabbr2	0.89	0.00	9.03	0.00

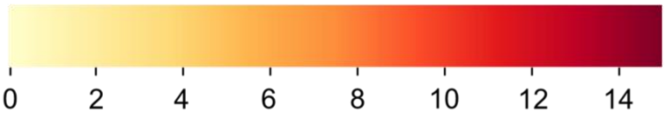

F) Glycine receptors

		Proteins	Genes	Sample 1	Sample 2	Sample 3	Sample 4
Glycine Subunits	α Subunits	α1	Glra1	0.00	6.78	7.00	5.07
		α2	Glra2	0.00	0.00	0.00	0.00
		α3	Glra3	0.00	0.00	0.00	0.00
		α4	Glra4	0.00	7.70	7.58	0.00
		β	Glrb	13.03	6.74	14.00	10.91

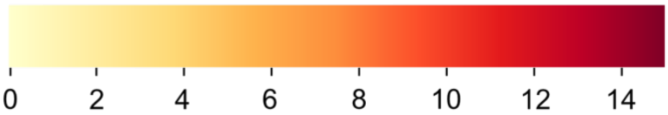

G) Ionotropic and metabotropic glutamatergic receptors

		Proteins	Genes	Sample 1	Sample 2	Sample 3	Sample 4
AMPA receptors	*Glu-R Subunits*	GluR1	Gria1	0.00	0.00	2.40	0.00
		GluR2	Gria2	0.00	6.65	10.19	12.08
		GluR3	Gria3	0.00	1.54	0.00	0.00
		GluR4	Gria4	0.00	0.97	10.85	10.18
	Delta	Gluδ1	Grid1	0.89	0.00	6.40	6.35
		Gluδ2	Grid2	0.89	0.00	9.12	5.48
Kainate receptors	*GluK Subunits*	GluK1	Grik1	0.00	0.00	10.13	1.60
		GluK2	Grik2	8.41	0.00	10.13	0.00
		GluK3	Grik3	0.00	0.00	1.05	8.40
		GluK4	Grik4	0.00	3.64	7.41	9.14
		GluK5	Grik5	0.00	8.81	9.16	9.27
NMDA receptors	*NR Subunits*	NR1	Grin1	5.86	0.00	9.31	3.02
		NR1os	Grin1os	0.00	0.00	1.65	0.00
		NR2a	Grin2a	0.00	1.54	0.00	5.11
		NR2b	Grin2b	0.89	1.54	1.65	2.83
		NR2c	Grin2c	0.00	0.00	0.00	0.00
		NR2d	Grin2d	5.39	4.26	5.93	5.91
		NR3a	Grin3a	0.00	0.97	0.00	0.00
		NR3b	Grin3b	0.00	5.18	0.00	0.00
		LFG1	Grina	14.48	14.87	14.44	14.37

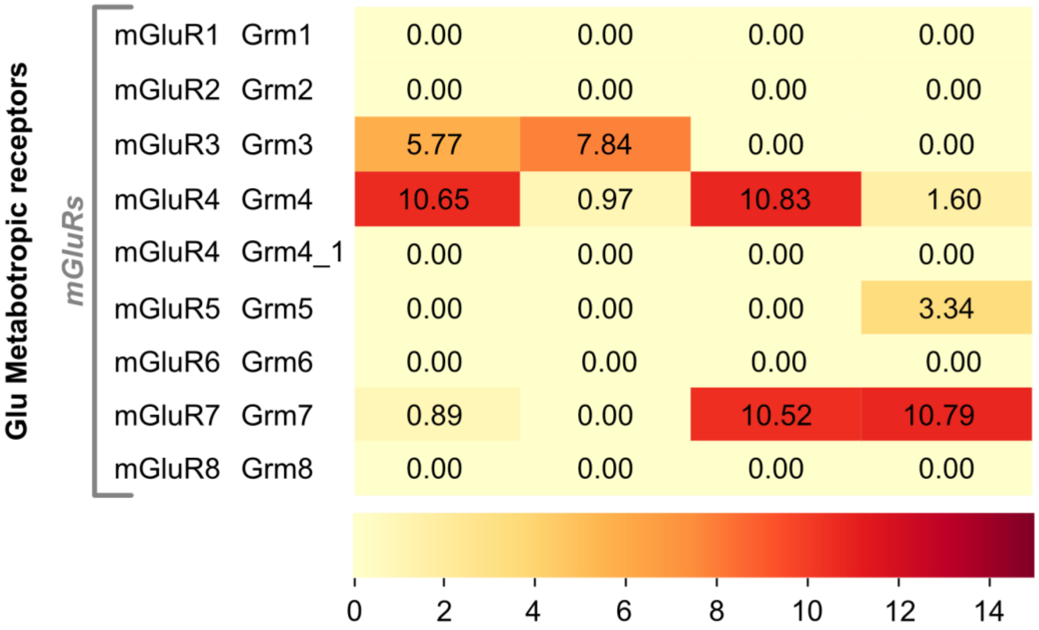
Glu Metabotropic receptors
mGluRs
mGluR1 Grm1 0.00 0.00 0.00 0.00
mGluR2 Grm2 0.00 0.00 0.00 0.00
mGluR3 Grm3 5.77 7.84 0.00 0.00
mGluR4 Grm4 10.65 0.97 10.83 1.60
mGluR4 Grm4_1 0.00 0.00 0.00 0.00
mGluR5 Grm5 0.00 0.00 0.00 3.34
mGluR6 Grm6 0.00 0.00 0.00 0.00
mGluR7 Grm7 0.89 0.00 10.52 10.79
mGluR8 Grm8 0.00 0.00 0.00 0.00
0 2 4 6 8 10 12 14

H) Ionotropic and metabotropic cholinergic receptors

Cholinergic receptors (ACh-Rs)	Subunits	Proteins	Genes	Sample 1	Sample 2	Sample 3	Sample 4
Nicotinic ACh-Rs (nACh-Rs)	α Subunits	α1	Chrna1	2.96	2.27	0.00	2.61
		α1os	Chrna1os	0.00	8.00	5.18	1.01
		α2	Chrna2	0.00	0.00	0.00	0.00
		α3	Chrna3	0.00	0.00	0.00	0.00
		α4	Chrna4	0.00	7.94	7.86	0.00
		α5	Chrna5	0.00	0.00	1.05	8.36
		α6	Chrna6	0.00	0.00	0.00	0.00
		α7	Chrna7	0.00	0.00	7.20	4.72
		α9	Chrna9	0.00	0.00	0.00	0.00
		α10	Chrna10	0.00	0.00	0.00	0.00
	β Subunits	β1	Chrnb1	0.00	0.00	0.00	1.01
		β2	Chrnb2	0.00	0.00	5.85	4.61
		β3	Chrnb3	0.00	0.00	0.00	0.00
		β4	Chrnb4	0.89	0.00	1.05	0.00
		δ	Chrnd	0.00	0.00	0.00	0.00
		ε	Chrne	6.55	0.00	5.71	0.00
		ε1	Chrne_1	6.55	0.00	5.71	0.00
Muscarinic ACh-Rs (mACh-Rs)		M1R	Chrm1	0.00	0.00	4.41	0.00
		M2R	Chrm2	1.43	1.54	11.59	7.59
		M3R	Chrm3	0.00	0.00	4.00	0.00
		M4R	Chrm4	0.00	0.00	0.00	0.00
		M5R	Chrm5	0.00	0.00	0.00	1.01

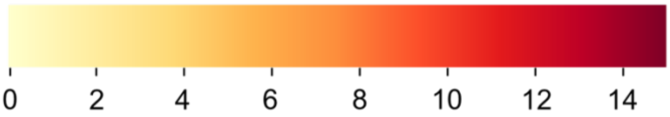

I) Ionotropic and metabotropic purinergic receptors

Purinergic receptors	Proteins	Genes	Sample 1	Sample 2	Sample 3	Sample 4
Ionotropic P2Xs	P2X1	P2rx1	6.81	9.01	9.25	8.11
	P2X1.1	P2rx1_1	0.00	7.99	8.04	7.65
	P2X2	P2rx2	0.00	0.00	0.00	0.00
	P2X3	P2rx3	0.00	6.65	3.41	0.00
	P2X4	P2rx4	12.92	13.53	12.88	13.59
	P2X5	P2rx5	0.00	0.00	0.00	0.00
	P2X5.1	P2rx5_1	0.00	0.00	0.00	0.00
	P2X6	P2rx6	1.43	0.00	0.00	0.00
	P2X7	P2rx7	13.01	13.28	12.51	13.09
Metabotropic P2Ys	P2Y1	P2ry1	0.00	0.00	0.00	0.00
	P2Y2	P2ry2	0.00	0.00	0.00	9.19
	P2Y4	P2ry4	0.00	0.00	0.00	0.00
	P2Y6	P2ry6	13.30	14.12	13.73	13.75
	P2Y10	P2ry10	0.00	0.00	0.00	0.00
	P2Y10b	P2ry10b	13.43	12.29	12.11	12.19
	P2Y12	P2ry12	19.52	19.51	19.34	19.34
	P2Y13	P2ry13	18.13	18.09	17.19	17.86
	P2Y14	P2ry14	0.00	1.54	0.00	0.00

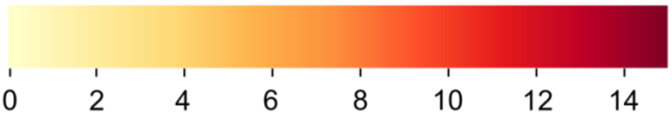

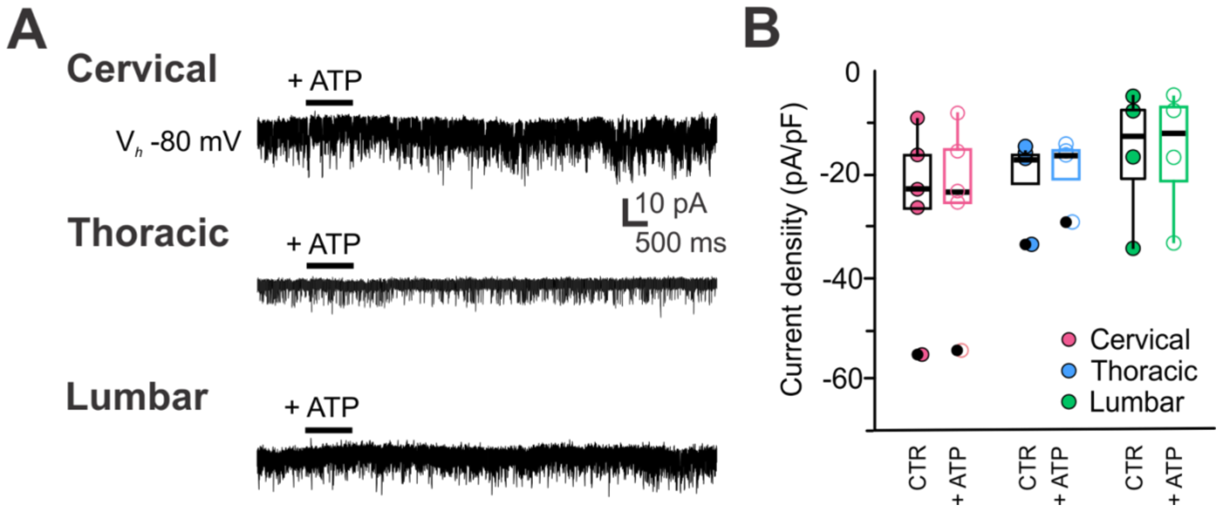

Supplementary Figure 4 - Along the central canal axis, CSF-cNs do not respond to ATP application.

A) Representative current traces recorded at V_h -80 mV in cervical, thoracic and lumbar CSF-cNs (***Top*** to ***Bottom***) upon pressure application of ATP-γ-S (a non-metabolized form of ATP, 100 µM for 1 s; black bar) to activate Purinergic ionotropic receptors (P_2Xs). **B)** Summary boxplots of the averaged current densities before and during ATP-γ-S application (mean of 500 ms recordings before and during application; + ATP, black bar) recorded at V_h -80 mV in CSF-cNs of the regions of interest (C: -28±18, -25±17 $pA.pF^{-1}$ (N=1, n=5); T: -20±9, -19±7 $pA.pF^{-1}$ (N=1, n=4) and L: -16±13, -16±13 $pA.pF^{-1}$ (N=1, n=5), data given for CTR and + ATP in order. *ANOVA.lme test*: F=0.4864, df=5 and 20, p(F)=0.7824 to compare currents densities between Conditions (CTR *vs.* ATP) and *Tukey (EMM) post-hoc test* to compare currents densities in the absence and presence of ATP within Regions (C: p=0/9558 (ns); T: 0.8772 (ns) and L: p=0.9744 (ns).

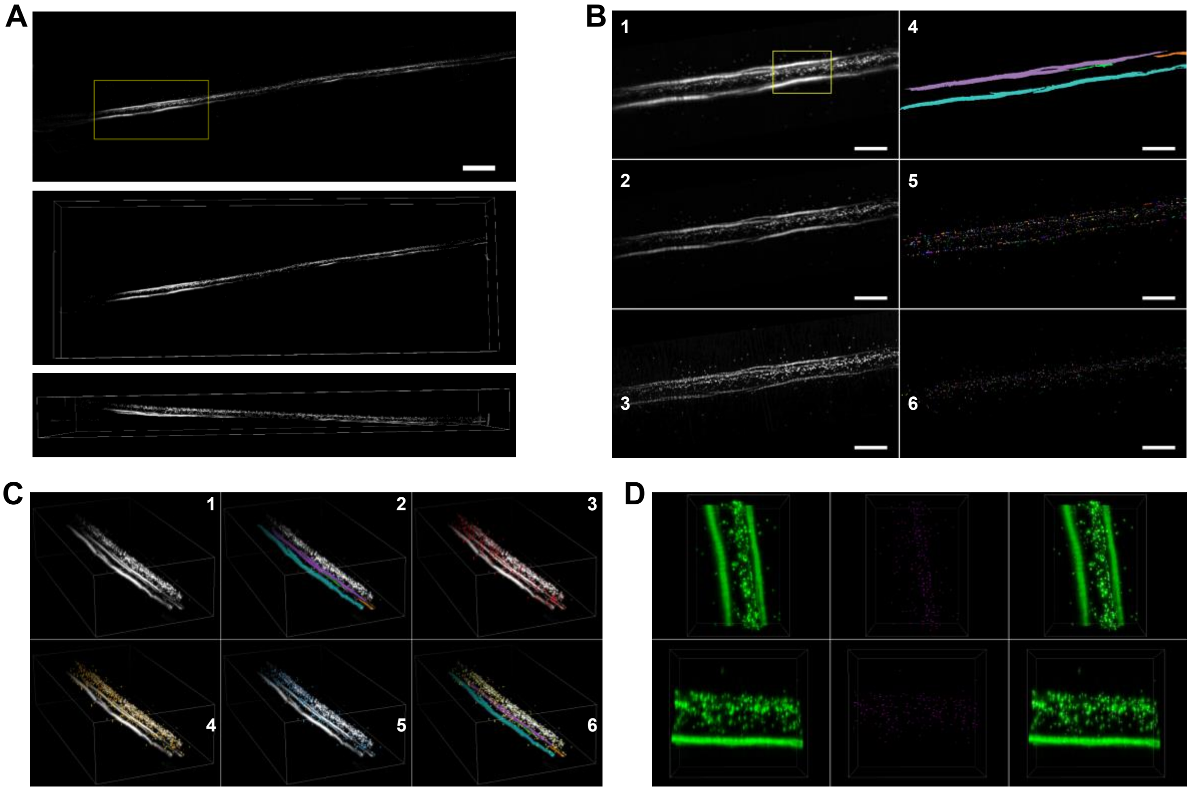

Supplementary Figure 5 - Workflow for cell segmentation and estimation of cell number.

A) Top. Maximal projection of a segment corresponding to the cervical SC of a PKD-tdTomato mouse. The inset box corresponds to the region depicted in Panel 1B. ***Middle*** and ***Bottom***, dorsal and lateral views, respectively of the 3D rendering for the region depicted in the ***Top*** Panel. Scale bar=500 µm. Dimensions of the box are (RC x LM x DV in µm) 7484.75 x 2632.50 x 841.75. **B)** Graphic workflow for image segmentation of the inset in Panel 1A (see methods). **1.** Maximal projection of the inset shown in Panel 1A. **2.** After rolling ball background subtraction r=10 pixels. **3.** After Difference of Gaussians filter (sigma1=1, sigma2=2). **4.** 3D objects corresponding to the ventromedial axon bundles of CSF-cNs. **5.** H-Maxima obtained from the image depicted in 3). **6.** H-Remaining particles after removing H-Maxima belonging to ventromedial axon bundles and filtering by volume and sphericity. Scale bar=200 µm. **C)** 3D representations of the views depicted in Panel 1B. **1.** Corresponds to Panel 1B1. **2.** Corresponds to Panel 1B4. **3.** corresponds to Panel 1B5. **4.** is Panel 1B5 after removing particles located in the ventral axon bundles. **5.** After filtering by volume. **6.** Merge of the final result of particle quantification and segmentation of the axon bundles. Dimensions of the box are (RC x LM x DV in µm) 1764.75 x 913.25 x 463.00. **D)** Inset of the box depicted in Panel 1B in dorsal (***Top***) and lateral (***Bottom***) views. ***Left.*** Fluorescence image of CSF-cNs. ***Middle.*** Centroids corresponding to particle analysis of this specific region. ***Right***. Merge of the centroids from particle analysis to the fluorescence microscopy images.

Table S1 - Composition of the intracellular solutions used for the sets of recording

Major Ion	KGlu (A)	CsAc-TEA (B)	KCl (C)	CsAc (D)
**Composition (mM)**				
**KCl**	3	-	130	-
**K-gluconate**	120	-	-	-
**NaCl**	5	5	10	5
**CsCl**	-	3	-	3
**CsAc**	-	100	-	120
**TEA-Cl**	-	20	-	-
**$MgCl_2$**	1	1	2	1
**$CaCl_2$**	1	0.5	1	1
**Free $[Ca^{2+}]_i$**	27 nM	12 nM	13 nM	12 nM
**EGTA**	2	-	5	10
**BAPTA**	-	10	-	-
**HEPES**	10			
**Phosphocreatine**	10			
**Mg-ATP**	4			
**Na_2-GTP**	0.2			
**pH**	7.35	7.35	7,35	7.34
**pH adjustment**	KOH 8N	CsOH 50% Wt/Vol	KOH 1M	CsOH 50% Wt/Vol
**Osmolarity**	~295 mOsmol.Kg^{-1}			
**Equilibrium potentials (mV) at 20-25°C**				
**E_{Na}**	+46	+46	+69	+44
**E_K**	-94	-	-97	/
**E_{Cl}**	-65	-64	+5	-59
**E_{Ca}**	+62	+65	+159	+107

Supplementary Table 2 - Composition of the extracellular solutions used to selectively isolate ionic conductances.

Experiments	Solution	Solution Label	Addition	Blockers
Characterization of intrinsic and AP discharge properties				
**Passive properties**	Intracellular	A-D<br>(A for CC)		
	Extracellular	aCSF		
Characterization of voltage-dependent channels				
**Na^+ Current**	Intracellular	B		
	Extracellular	Na_V	+TEA (20mM)<br>without $CaCl_2$<br>replaced by 4mM $MgCl_2$	TTX (0,5 µM) puff
**K^+ Current**	Intracellular	A		
	Extracellular	K_V	+TTX (0,5 µM)<br>without $CaCl_2$<br>replaced by 4mM $MgCl_2$	TEA (10 mM) bath<br>+4-AP (4 mM) puff
**Ca^{2+} Current**	Intracellular	B		
	Extracellular	Ca_V	+TTX (0,5 µM)<br>+TEA (20mM)	Cadmium (200 µM)<br>bath and/or puff
Characterization of ionotropic ligandd-gated receptors				
**GABA (1mM)**	Intracellular	C		
	Extracellular	aCSF	+TTX (0.5 µM)<br>+Stry (1 µM)<br>+D-tubocurarine<br>+DNQX (20 µM)	Gbz (10 µM)<br>+Picrotoxine (100 µM)
**Glycine (1mM)**	Intracellular	C		
	Extracellular	aCSF	+TTX (0.5 µM)<br>+Gbz (10 µM)<br>+D-tubocurarine<br>+DNQX (20 µM)	Stry (1µM)
**Glutalmate AMPA ; NMDA ; Kainate (100µM)**	Intracellular	C (VC) / A (CC)		
	Extracellular	aCSF	***<u>Voltage-Clamp</u> :***<br>+TTX (0.5 µM)<br>+Gbz (10µM)<br>+Picrotoxine (100 µM)<br>+Stry (1 µM)<br>+D-tubocurarine (100µM)<br>***<u>Current-Clamp</u> :***<br>+Gbz (10µM)<br>+Picrotoxine (100 µM)<br>+Stry (1 µM)<br>+D-tubocurarine (100µM)	DNQX (400µM)

**ACh** **(4 mM)**	Intracellular	D (VC) / A (CC)		
	Extracellular	aCSF	***<u>Voltage-Clamp</u> :***<br>+Atropine (10 µM)<br>+TTX (0.5 µM)<br>+Gbz (10µM)<br>+Picrotoxine (100 µM<br>+DNQX (400µM)<br>***<u>Current-Clamp</u> :***<br>+Atropine (10 µM)<br>+Gbz (10µM)<br>+Picrotoxine (100 µM<br>+DNQX (400µM)<br>+D-tubocurarine (100µM)	D-tubocurarine (100µM)
**ATP-γ-S** **(1 mM)**	Intracellular	C		
	Extracellular	aCSF	+TTX (0.5 µM)<br>+Gbz (10µM)<br>+Picrotoxine (100 µM<br>+Stry (1 µM)<br>+DNQX (400µM)	
GPCR modulation of Ca_V				
**Baclofen** **(100 µM, 40s)**	Intracellular	B		
	Extracellular	Ca_V Modulation	TTX (0.5 µM)<br>+ TEA (20 mM)<br>+ Strych (1µM<br>+ DNQX (20µM)<br>+ Gbz (10µM)	CGP (2 µM)
**OxoM** **(100µM, 50s)**	Intracellular	B		
	Extracellular	Ca_V Modulation	TTX (0.5 µM)<br>+ TEA (20 mM)<br>+ Strych (1µM)<br>+ DNQX (20µM)<br>+ Gbz (10µM)	Atropine (10 µM)
