# Supplementary material for: Cerebrospinal fluid-contacting neurons are sensory neurons with uniform morphological and region-specific electrophysiological properties in the mouse spinal cord
Source: Commun Biol. 2025 Aug 15;8:1233. doi: 10.1038/s42003-025-08559-x (PMC12356878; doi:10.1038/s42003-025-08559-x)
Supplement: Supplementary file 3 — Reporting Summary [file 42003_2025_8559_MOESM3_ESM.pdf]

nature portfolio

Corresponding author(s): Prof. Nicolas Wanaverbecq
COMMSBIO-24-7972D

Last updated by author(s): Jun 28, 2025

Reporting Summary

Nature Portfolio wishes to improve the reproducibility of the work that we publish. This form provides structure for consistency and transparency in reporting. For further information on Nature Portfolio policies, see our Editorial Policies and the Editorial Policy Checklist.

Statistics

For all statistical analyses, confirm that the following items are present in the figure legend, table legend, main text, or Methods section.

n/a	Confirmed	
☐	☒	The exact sample size (n) for each experimental group/condition, given as a discrete number and unit of measurement
☐	☒	A statement on whether measurements were taken from distinct samples or whether the same sample was measured repeatedly
☐	☒	The statistical test(s) used AND whether they are one- or two-sided<br>*Only common tests should be described solely by name; describe more complex techniques in the Methods section.*
☒	☐	A description of all covariates tested
☐	☒	A description of any assumptions or corrections, such as tests of normality and adjustment for multiple comparisons
☐	☒	A full description of the statistical parameters including central tendency (e.g. means) or other basic estimates (e.g. regression coefficient) AND variation (e.g. standard deviation) or associated estimates of uncertainty (e.g. confidence intervals)
☐	☒	For null hypothesis testing, the test statistic (e.g. F, t, r) with confidence intervals, effect sizes, degrees of freedom and P value noted<br>*Give P values as exact values whenever suitable.*
☒	☐	For Bayesian analysis, information on the choice of priors and Markov chain Monte Carlo settings
☒	☐	For hierarchical and complex designs, identification of the appropriate level for tests and full reporting of outcomes
☒	☐	Estimates of effect sizes (e.g. Cohen's d, Pearson's r), indicating how they were calculated

Our web collection on statistics for biologists contains articles on many of the points above.

Software and code

Policy information about availability of computer code

Data collection	Imaging data were collected using:<br><br>Electrophysiological were acquired and analyzed using the pClamp 9_11 (ClampEx and ClampFit) suite (Molecular Device, Inc.).
Data analysis	Data were processed and analyzed using R studio software (Version 2024.04.2+764 (2024.04.2+764)), Image J , Origin Pro and GraaaphPad Prism (V.)

For manuscripts utilizing custom algorithms or software that are central to the research but not yet described in published literature, software must be made available to editors and reviewers. We strongly encourage code deposition in a community repository (e.g. GitHub). See the Nature Portfolio guidelines for submitting code & software for further information.

Data

Policy information about availability of data

All manuscripts must include a data availability statement. This statement should provide the following information, where applicable:

- Accession codes, unique identifiers, or web links for publicly available datasets
- A description of any restrictions on data availability
- For clinical datasets or third party data, please ensure that the statement adheres to our policy

The data of this manuscript are available from the corresponding author upon reasonable request or can be found at Figshare depositrory: DOI 10.6084/m9.figshare.29430869

Human research participants

Policy information about studies involving human research participants and Sex and Gender in Research.

Reporting on sex and gender	Not applicable
Population characteristics	Not applicable
Recruitment	Not applicable
Ethics oversight	Not applicable

Note that full information on the approval of the study protocol must also be provided in the manuscript.

Field-specific reporting

Please select the one below that is the best fit for your research. If you are not sure, read the appropriate sections before making your selection.

☒ Life sciences ☐ Behavioural & social sciences ☐ Ecological, evolutionary & environmental sciences

For a reference copy of the document with all sections, see nature.com/documents/nr-reporting-summary-flat.pdf

Life sciences study design

All studies must disclose on these points even when the disclosure is negative.

Sample size	Typically we considered that 3 animals were sufficient to describe anatomical data and for electrophysiological analysis a minimum of 10 were considered collected for more than 2 animals and 3 slices
Data exclusions	Not applicable
Replication	Each experiments was repeated in a minimum of 3 animals to ensure replication and electrophysiological recordings collected from at least 10 cells in each set of experiments. Individuala data points are presented in all graphs.
Randomization	Not applicable
Blinding	Not applicable

Reporting for specific materials, systems and methods

We require information from authors about some types of materials, experimental systems and methods used in many studies. Here, indicate whether each material, system or method listed is relevant to your study. If you are not sure if a list item applies to your research, read the appropriate section before selecting a response.

Materials & experimental systems

n/a	Involved in the study	
☐	☒	Antibodies
☒	☐	Eukaryotic cell lines
☒	☐	Palaeontology and archaeology
☐	☒	Animals and other organisms
☒	☐	Clinical data
☒	☐	Dual use research of concern

Methods

n/a	Involved in the study	
☒	☐	ChIP-seq
☒	☐	Flow cytometry
☒	☐	MRI-based neuroimaging

Antibodies

Antibodies used	Not applicable
Validation	Not applicable

Animals and other research organisms

Policy information about studies involving animals; ARRIVE guidelines recommended for reporting animal research, and Sex and Gender in Research

Laboratory animals	We used wild type C57 Black6J (Charles River), PKD2L1-Cre (Pkd2l1tm1(cre); MGI ID: 6451758; a generous gift Emily Leman), and Choline Acetyl Transferase-Cre (ChAT-Cre; Chattm2(cre)Lowl; The Jackson Laboratory, MGI ID: 5475195) mice. PKD2L1- and ChAT-Cre animals were cross-breed with flex-tdTomato mice (Gt(ROSA)26Sortm14 (CAG-tdTomato)Hze, The Jackson Laboratory; MGI ID: 3809524) to generate PKD2L1-Cre (PKD-tdTomato)- and ChAT (ChAT-tdTomato)-Cre::flex-tdTomato mice and to selectively express the tdTomato fluorescent protein in the neuronal population of interest. Animal of either sex were used for histology as well as for electrophysiological recordings (3-6 Weeks old mice).<br>We also performed icv injection of viral constructs in ChAT-tdTomato (ChAT-Cre; Chattm2(cre)Lowl; The Jackson Laboratory, MGI ID: 5475195 crossed with flex-tdTomato mice (Gt(ROSA)26Sortm14 (CAG-tdTomato)Hze, The Jackson Laboratory; MGI ID: 3809524).
Wild animals	Not applicable
Reporting on sex	Experiments were conducted on both sex and no gender based difference waas observed
Field-collected samples	Not applicable
Ethics oversight	All experiments were conducted in conformity with the rules set by the EC Council Directive (2010/63/UE) and the French “Direction Départementale de la Protection des Populations des Bouches-du-Rhône (DDPP13)” (Project License Nr: APAFIS 44331,2023071917567777 & 33336, 2021101819022439. NW and License for the Use of Transgenic Animal Models Nr: DUO-5214). Protocols used agree with the rules set by the Comité d'Ethique de Marseille (CE71), our local Committee for Animal Care and Research.

Note that full information on the approval of the study protocol must also be provided in the manuscript.
